# Progression of QRS duration – a potential surrogate marker of survival in ATTRwt amyloidosis patients

**DOI:** 10.1186/s13023-025-04078-4

**Published:** 2025-10-20

**Authors:** Selina Hein, Fabian aus dem Siepen, Sonja M. Zierleyn, Maximilian Knoll, Hugo A. Katus, Norbert Frey, Arnt V. Kristen

**Affiliations:** 1 Department of Cardiology, Angiology and Pulmonology, University Hospital Heidelberg, Heidelberg, Germany; 2 DZHK (German Center for Cardiovascular Research), Site Heidelberg/Mannheim, Heidelberg, Germany; 3 Department of Radiation Oncology, Heidelberg Ion-Beam Therapy Center (HIT), Heidelberg University Hospital (UKHD), German Cancer Research Center, Heidelberg, Germany; 4 German Cancer Consortium (DKTK) Core-Center, German Cancer Research Center (DKFZ), 69120 Heidelberg, Germany; 5 Divisions of Molecular & Translational Radiation Oncology, Heidelberg Faculty of Medicine (MFHD) and Heidelberg University Hospital (UKHD), Heidelberg Ion-Beam Therapy Center (HIT), 69120 Heidelberg, Germany; 6 Heidelberg Institute of Radiation Oncology (HIRO), National Center for Radiation Research in Oncology (NCRO), German Cancer Research Center (DKFZ) and Heidelberg University Hospital (UKHD), 69120 Heidelberg, Germany; 7 CCU Translational Radiation Oncology, National Center for Tumor Diseases (NCT), German Cancer Research Center (DKFZ) and Heidelberg University Hospital (UKHD), 69120 Heidelberg, Germany

**Keywords:** Routine care, Survival, Transthyretin, Wild-type ATTR amyloidosis

## Abstract

**Introduction:**

In wild-type transthyretin amyloidosis (ATTRwt), the deposition of transthyretin in the myocardium leads to progressive heart failure. However, little is known about the short-term natural progression of this disease and potential predictors of outcome.

**Objectives & methods:**

Therefore, this study longitudinally analyzed the clinical findings (ECG, echocardiography, and laboratory tests) of 65 patients suffering from ATTRwt at baseline and at follow-up visits after 12 months.

**Results:**

In total, 44 patients (67.7%) presented with abnormal ECGs, prolonged PR and QRS durations and low-voltage patterns. Pacemaker placement was performed in eleven patients (16.9%). At the one-year follow-up visit, we detected significant increases in the QRS duration (from 119.7 ± 4.0 to 125.9 ± 4.3 ms; *p* < 0.05), intraventricular septum thickness (from 18.9 ± 0.5 mm to 19.9 ± 0.5 mm; *p* < 0.05) and ejection fraction (EF) (from 45.4 ± 2.1% to 38.9 ± 2.6%; *p* < 0.05) compared with those at the baseline visit. During follow-up, 16 patients (24.6%) died. Predictors of worse outcomes were progression in QRS duration, low EF, reduced renal function, impaired right ventricular function and the need for pacemaker implantation.

**Conclusions:**

In ATTRwt, precise evaluation via routine cardiac diagnosis helps to identify patients with an elevated risk of mortality. Patients with prolonged QRS duration, progressive myocardial hypertrophy, right ventricular failure, reduced renal function and the need for cardiac pacemakers are at increased risk for one-year mortality.

**Supplementary Information:**

The online version contains supplementary material available at 10.1186/s13023-025-04078-4.

## Introduction

Amyloidosis comprises a group of rare diseases caused by the deposition of diverse physiological proteins in tissues, resulting in progressive organ dysfunction. Transthyretin (TTR) is one of the proteins that most frequently causes cardiac amyloidosis. As a homotetramer, this protein is the physiological transporter of thyroxin and vitamin A in the blood. Two distinct disease entities exist in ATTR amyloidosis. In variant ATTR (ATTRv) amyloidosis, an individual point mutation in the *TTR* gene leads to structural changes and reduced stability of the TTR tetramer, causing sensomotoric neuropathy and (restrictive) cardiomyopathy. However, wild-type ATTR (ATTRwt) amyloidosis occurs without any pathogenic variants in the TTR gene. It typically affects the heart of male individuals older than 60 years of age. In both ATTR entities amyloid deposition in the myocardium typically lead to myocardial fibrosis, restrictive cardiomyopathy and heart failure. In ECG bundle branch blocks, low voltage patterns and Q-waves are commonly described in cardiac amyloidosis. Interestingly, direct infiltration of the conduction system is histologically rarely found in ATTRwt patients therefore conduction disturbances are supposed to occur secondary due to myocardial fibrosis surrounding amyloid deposits [1–3]. These conduction disturbances result in various rhythm and conduction abnormalities whose clinical impact has not yet been broadly evaluated.

In recent years, improvements in diagnostic techniques, especially strain measurements, cardiac MRI and ^99m^Tc-DPD scintigraphy, have significantly increased the rate of patients newly diagnosed with ATTRwt amyloidosis [4]. Analyzation of study cohorts from the US and UK revealed NTproBNP, hsTNT and GFR as potent risk prediction parameters for mortality [5–8]. However, these parameters may fluctuate due to changes in renal function or fluid status, limiting their short-term utility. In cardiac AL Amyloidosis this limitation was addressed by calculating ECV in cardiac MRI as a surrogate for cardiac amyloid load [9]. However, compared to MRI, ECGs are widely available, easy and quick to record and inexpensive.

As a standardized and objective metric, QRS duration offers little interobserver variability and can be reliably assessed across different clinical settings. Pathophysiologically, QRS widening in ATTRwt may reflect progressive myocardial infiltration and fibrosis due to amyloid deposition, rather than isolated conduction system involvement. However, the prognostic significance of increasing QRS duration over time has not yet been systematically investigated.

Therefore, this study investigates short-term changes in electrocardiographic and echocardiographic parameters in patients with ATTRwt and tries to determine whether progression of QRS duration is associated with worse outcomes.

We thereby hope to contribute the developement evidence-based tools for rhythm monitoring and therapeutic regimens in ATTRwt. As disease-specific therapies for ATTR cardiomyopathy are meanwhile available, precise knowledge about 12-month disease development and risk predictors will help clinicians detect treatment response and disease progression, enabling more effective use of these costly treatments.

## Methods

### Study population

This study included 65 patients suffering from ATTRwt amyloidosis who presented at a tertiary referral center for amyloidosis at Heidelberg University Hospital between 2014 and 2015. None of the patients received any causative treatment, namely, TTR stabilizers or silencers. The data were analyzed retrospectively as approved by the ethical review committee Heidelberg (S186-2013). All patients were diagnosed with ATTRwt amyloidosis either by endomyocardial biopsy or ^99m^Tc-DPD scintigraphy, a typical amyloidosis pattern on cardiac MRI and the exclusion of a pathogenic variant in the TTR gene. Electrocardiography (ECG), echocardiography, laboratory tests and clinical symptoms were derived from the patients’ medical records. A follow-up visit between 9 and 15 months after the baseline visit was accepted. The mean time between the baseline visit and the follow-up visit was 13.6 ± 1.43 months. The mean follow-up period for survival analyses was 61.3 ± 5.8 months.

### Clinical data

Patient history and physical examination data, including the Karnofsky index, New York Heart Association (NYHA) class, body size and weight, were recorded for all patients. Body surface area (BSA), body mass index (BMI) and modified body mass index (mBMI) were calculated. To attain mBMI, BMI was multiplied by plasma albumin levels to correct for potential fluid retention due to hypalbuminemia. Moreover, episodes of cardiac decompensation and pacemaker placement during the observation period were recorded. Finally, the incidence of comorbidities, including stroke, carpal tunnel syndrome (CTS), coronary artery disease and diabetes, was noted.

### Laboratory tests

At baseline and follow-up visits, our healthcare staff took blood samples from a venipuncture and sent them directly to the accredited laboratory for analysis.

### Instrumental diagnostics

#### Electrocardiogram (ECG)

From a routine ECG, heart rhythm, heart rate, PR, the QT interval, and QRS duration were analyzed. The corrected QT time was calculated via the Bazett formula (QTc = QT(ms)√RR distance) [10]. Furthermore, QRS abnormalities, namely, Q spikes or bundle branch blocks, were documented. A low voltage pattern was defined as a QRS amplitude ≤0.5 mV in all limb leads or ≤1 mV in all precordial leads or [11] if the sum of the R amplitude in leads V1/2 and the S amplitude in leads V5/6 were less than 1 mV [12]. ECGs showing continuous ventricular pacing at baseline or follow-up visit were excluded from the detailed analysis of ECGs.

#### Echocardiography

Echocardiography records were analyzed retrospectively. The left ventricular (LV) mass was derived from the LV end-diastolic diameter (LVEDD), the thickness of the posterior wall and the intraventricular septum (IVS) via the Devereux formula (LV mass = 1.05*((LVEDD+posterior wall+IVS)^3^ - (LVEDD)^3^))/1000). The LV mass index was attained by dividing the LV mass through the BSA. The end-diastolic volume (EDV) and end-systolic volume (ESV) were calculated (EDV = 4.5*LVEDD^2^/100 and ESV = 4.5*LVESD^2^/100). Cardiac stroke volume (SV) was attained through calculation of the difference between the two volumes. The LV ejection fraction (LV-EF) was calculated according to the following formula: LV-EF = SV/EDV [13]. The myocardial volume was calculated as the ratio of the LV mass to the specific myocardial mass (1.05 g/ml). The myocardial contraction fraction (MCF) was calculated from the stroke volume (SV) divided by the myocardial volume. For diastolic function, pulse wave Doppler measurements at the tips of the mitral valve were analyzed to obtain E- and A-wave amplitudes, and tissue Doppler measurements (TD-I) at the septal and lateral annuli of the mitral valve were conducted to obtain e’- and a’-waves. E-wave deceleration time (DTe) and E/A and E/e’ quotients were calculated and classified into the following groups: no diastolic dysfunction, relaxation dysfunction (A > E, E/e` < 8), pseudonormalization (E/e` between 8 and 15) and distinct diastolic dysfunction (E/e` > 15). Finally, mitral annular plane systolic excursion (MAPSE) and tricuspid annular plane systolic excursion (TAPSE) were measured via M-Mode through the lateral mitral and lateral tricuspid annuli.

### Statistics

Statistical analyses were conducted via SPSS and R. For descriptive statistics means and SEM were calculated. Differences between groups were calculated via the Mann‒Whitney U test for continuous variables. Correlation analyses were conducted using Pearson’s correlation. Time-to-event analyses were conducted with Cox-PH models and parameteric survival models assuming a Weibull distribution with the survival package [14]. Optimal separation of groups was identified by minimizing the p-value for varying cutoffs using the dataAnalysisMisc package [15]. Significance level alpha was set to 0.05 (two-sided). Graphical data presentation were conducted using R, v3.6.3 [16].

## Results

### Patient characteristics and demographics

We retrospectively analyzed data from 65 patients diagnosed wild-type tranthyretin amyloidosis (ATTRwt) at a German tertiary referral center at their initial visit and at their first follow-up visit after 12 months. The mean age was 73.7 ± 0.7 years, and 92.3% were male. The diagnosis was confirmed by either endomyocardial biopsy or 99mTc-DPD scintigraphy, in accordance with current European guidelines [17].

At presentation, 73.4% (*n* = 47) reported cardiac symptoms, predominantly exertional dyspnea (NYHA II/III, *n* = 45; NYHA IV, *n* = 2). The remaining 26.2% had non-cardiac manifestations, including polyneuropathy in 11 patients (16.9%). In most patients, the polyneuropathy was evidently unrelated to amyloidosis. In only one of these patients (1.5%), the diagnosis was not clear; therefore, the person underwent nerve biopsy without detection of neural ATTR deposition. Ten patients (15.4%) had pre-existing cardiac pacemakers at baseline.

### Baseline visit: ECG and echocardiography

**Fig. 1:**
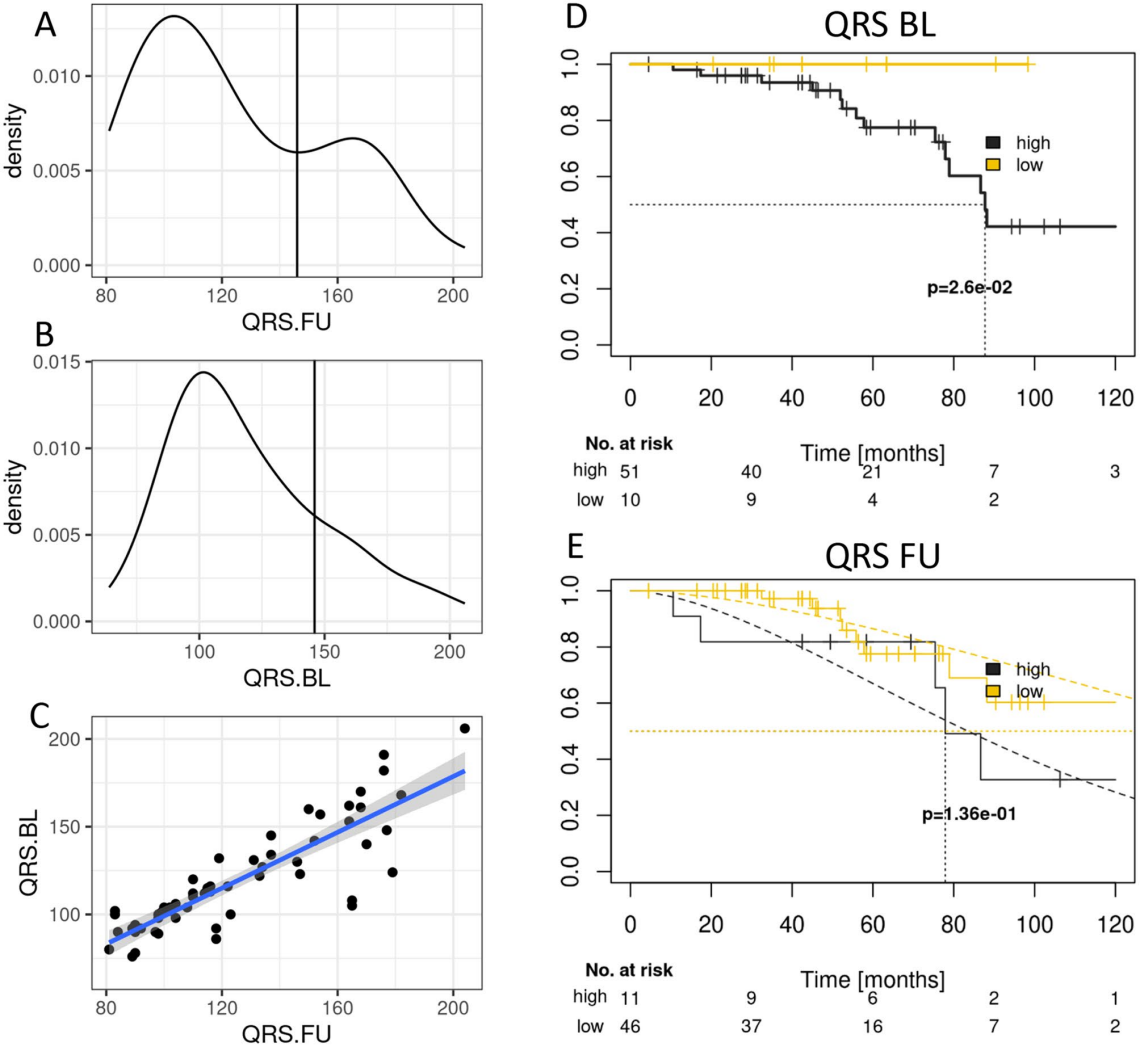
QRS duration at follow up visit (FU) in ms and its frequency density (**A**). QRS duration at baseline visits (BL) in ms and its frequency density (**B**). Correlation of QRS duration at FU with QRS duration at BL (**C**). Kaplan Meier curves of survival probability in accordance to low and high QRS duration and its p-value (**D**), Kaplan Meier survival curves of high and low QRS durations at FU in accordance to follow-up times in months (**E**)

At baseline, ECG abnormalities were observed in 67.7% of patients, including prolonged PR (mean: 201.5 ± 7.4 ms) and QRS durations (mean: 119.7 ± 4.0 ms), pathological Q-waves (18.5%) and low-voltage patterns (12.3%). Atrial fibrillation was present in 43.1% (see Table 1). Seven ECGs presenting continuous ventricular pacing, necessitating their exclusion from further evaluation. At baseline the range of QRS durations was 64 to 206 ms and QRS duration density analysis showed a single peaked distribution (see Fig. 1B).

**Fig. 2:**
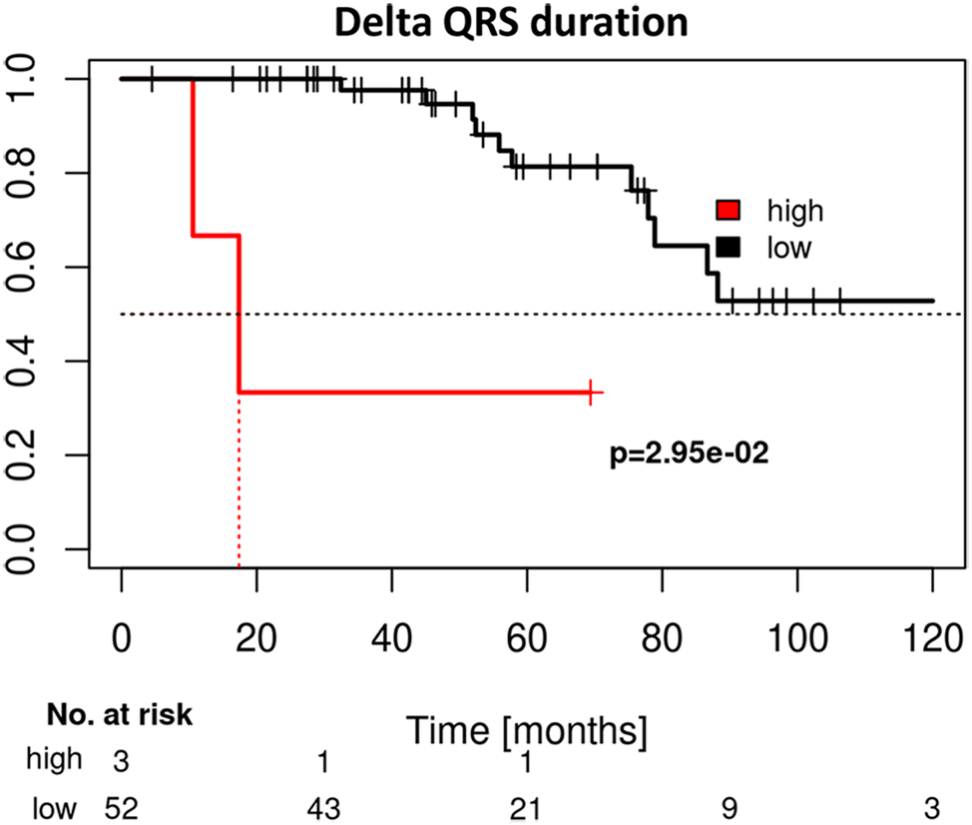
Kaplan Meier curves presenting the predictive value of Delta QRS duration between baseline (BL) and follow-up visits (FU) for patient with high and low differences in QRS durations and their probability of survival

**Table 1:** Study cohort characteristics

Patient characteristics	Value (± SEM or %)
Age (years)	73.7 ± 0.7
Male	60 (92.3%)
Female	5 (7.7%)
Comorbidities	
Arterial hypertension	45 (69.2%)
Coronary artery disease	16 (24.6%)
Cardiac decompensation	8 (12.3%)
**ECG**	
Heart rate (bpm)	71.4 ± 1.7
Abnormal ECG	44 (67.7%)
PR time (ms)	201.5 ± 7.4
QRS-duration (ms)	119.7 ± 4.0
QRS > 130 ms	20 (30.7%)
Pathological Q-waves	12 (18.5%)
Low voltage pattern	8 (12.3%)
Atrial fibrillation	28 (43.1%)
**Echocardiography**	
Left atrial diameter (mm)	45.0 ± 0.9
Intraventricular septum (mm)	18.9 ± 0.5
Posterior wall (mm)	16.3 ± 0.5
MAPSE (cm)	1.0 ± 0.5
TAPSE (cm)	1.4 ± 0.1
Myocardial mass (g/m^2^)	218.2 ± 10.1
Enddiastolic diameter (mm)	45.8 ± 0.8
Endsystolic diameter (mm)	32.5 ± 1.0
Enddiastolic volume (ml)	87.9 ± 3.8
Endsystolic volume (ml)	48.1 ± 3.2
E/e’	12.8 ± 0.7

Parameters of the study population and baseline visits are depicted as mean±standard error of mean (SEM), the other values are depicted as number of individuals and percentage of study cohort

Echocardiography revealed structural and functional signs of cardiac amyloidosis: increased intraventricular septal (IVS) thickness (18.9 ± 0.5 mm), elevated LV mass index (218.2 ± 10.1 g/m²), and impaired longitudinal function (TAPSE 1.4 ± 0.1 cm; MAPSE 1.0 ± 0.5 cm). Diastolic dysfunction was common (mean E/e’: 12.8 ± 0.7), while LVEF was moderately reduced (45.5 ± 2.1%).

### Follow-up examinations after 12 months

Sixty patients (92.3%) presented for the 12-month follow-up visit. Compared to baseline, significant ECG changes were observed: QRS duration increased by 6.2 ± 2.2 ms (*p* < 0.05), bundle branch block prevalence rose from 47.7% to 66.1% (*p* < 0.05), pathological Q-waves increased from 18.5% to 55.9% (*p* < 0.05) (see Table 2). During the follow-up period, five patients received cardiac pacemakers. Of those, two presented with continuous ventricular pacing on their ECGs and were therefore excluded from further analysis. Remarkably, at follow-up visits the QRS duration range raised to 92 until 204 ms and QRS duration density analysis revealed a two peaked distribution of QRS durations suggesting a subgroup progression of QRS duration (see Fig. 1A).

**Table 2:** Comparison of the incidence of ECG findings between baseline and 1 year follow-up visits

Characteristics	BL (%)	1-year FU (%)	*p*-value
Abnormal ECG	67.7	88.1	*p* < 0.05
Sinus rhythm	56.9	54.2	n.s.
q-wave	18.5	55.9	*p* < 0.05
Bundle block	47.7	66.1	*p* < 0.05
QRS-duration > 130 ms	30.7	39	n.s.
Low voltage pattern	12.3	6.8	n.s.

Prevalence of ECG findings is indicated as % from all study participants, BL: baseline FU: follow-up

Echocardiography showed subtle but statistically significant morphological and functional progression: IVS thickness increased by +1.0 ± 0.3 mm (*p* < 0.05), LVEF declined by −6.7 ± 3.1% (*p* < 0.05), stroke volume and myocardial contraction fraction (MCF) decreased, while left atrial diameter increased by +0.2 ± 0.01 mm (*p* < 0.05) (see Table 3). In terms of biomarkers no significant differences between baseline (BL) and follow-up (FU) visits were detected (see Table 3).

**Table 3:** ECG, echocardiography and laboratory test at follow-up examination

Parameter	Baseline	Delta at 1-year FU	*p*-value
**zECG**			
Heart rate (1/min)	73.5 ± 1.7	2.1 ± 1.8	n.s.
PR time (ms)	199.7 ± 6.2	−1.9 ± 5.1	n.s.
QRS-duration (ms)	119.7 ± 4.0	6.2 ± 2.2	*p* < 0.05
QTC time (ms)	449.0 ± 0.5	0.0 ± 3.6	n.s.
**Echocardiography**			
**Morphology**			
Left atrium (mm) IVS (mm) PW (mm) LVEDD (mm) LVESD (mm) EDV (ml) ESV (ml) EF (%) LV mass devereaux myocardial volume (ml) Myocardial volume (ml) Stroke volume (ml) MCF (%)	43.0 ± 0.8 18.9 ± 0.5 16.9 ± 0.5 43.0 ± 0.9 32.3 ± 0.9 86.8 ± 3.2 45.3 ± 3.2 45.5 ± 2.1 236.7 ± 8.3 225.5 ± 7.9 39.7 ± 2.0 0.2 ± 0.01	−1.9 ± 0.9 +1.0 ± 0.3 0.7 ± 0.4 −0.8 ± 0.8 −0.1 ± 1.0 −1.2 ± 3.4 −2.8 ± 3.5 −6.7 ± 3.1 18.5 ± 9.3 17.7 ± 8.9 −6.2 ± 2.7 −0.1 ± 0.01	*p* < 0.05 *p* < 0.05 n.s. n.s. n.s. n.s. n.s. *p* < 0.05 n.s. n.s. *p* < 0.05 *p* < 0.05
**Myocardial function**			
A (m/s)	0.23 ± 0.04	−0.08 ± 0.03	*p* < 0.05
E (m/s)	0.8 ± 0.0	0.0 ± 0.0	n.s.
E/A	2.0 ± 0.2	0.2 ± 0.2	n.s.
E/e’	13.0 ± 0.9	0.8 ± 1.0	n.s.
MAPSE (cm)	0.9 ± 0.5	−0.1 ± 0.1	n.s.
TAPSE (cm)	1.4 ± 0.1	−0.3 ± 0.1	*p* < 0.05
**Laboratory tests**			
*Cardiac biomarker* hsTNT (pg/ml) NT-proBNP (ng/l)	45.4 ± 2.5 3962.7 ± 412.0	−1.2 ± 5.2 −85.0 ± 351.9	n.s. n.s.
*Renal function* Creatinine (mg/dl) MDRD (ml/min *1.73qm) Urea (mg/dl)	1.3 ± 0.1 62.1 ± 3.7 49.4 ± 3.9	0.2 ± 0.0 1.1 ± 3.0 −8.3 ± 3.8	n.s. n.s. *p* < 0.05

Values are depicted as means±standard error of mean (SEM) and differences between baseline and follow-up visits. LVEDD: left ventricular enddiastolic diameter, LVESD: left ventricular endsystolic diameter, EDV: enddiastolic volume, ESV: endsystolic volume, LVmass Devereaux BSA: left ventricular mass calculated according to the Devereaux formular indexed to the body surface area, TDI: tissue Doppler imaging, DT: deceleration time, MAPSE: mitral annular plane systolic excursion. hsTNT: high sensitive troponin T. PW: posterior wall, NT-proBNP: N-terminal pro brain natriuretic peptide, MDRD: modification of diet in renal disease, formula to calculate glomerular filtration rate

### Pearson’s correlation of QRS duration with echocardiographic and laboratory parameters

At both baseline and follow-up, QRS duration showed moderate positive correlations with: Myocardial volume (*r* = 0.4, *p* = 0.035, left atrial size (*r* = 0.3, *p* = 0.03) and a negative correlation with TAPSE (*r* = −0.3, *p* = 0.015), suggesting its association with both morphological and functional cardiac deterioration (see Table 4). Furthermore, correlation analyses revealed positive correlation of QRS durations at baseline (BL) and follow-up (FU) visits (see Fig. 1C).

**Table 4:** Correlation parameters of ECG

Parameters	Correlation coefficient	*p*-value
**Baseline visits**		
QRS-duration vs myocardial volume	0.4	0.035
QRS-duration vs hsTNT	0.27	0.048
QRS- vs QTc-duration	0.7	< 0.001
QRS-duration vs TAPSE	−0.3	0.015
QRS-duration vs LA	0.3	0.030
Myocardial volume vs QTc-duration	0.4	0.004
**Follow-up examination**		
QRS-duration vs LA	0.3	0.03
QRS- vs QTc-duration	0.6	< 0.001
IVS vs hsTNT	0.39	0.023
Stroke volume vs IVS	−0.3	0.012

Echocardiography and laboratory test are depicted. LA: left atrium, IVS: intraventricular septum. Pearson’s correlations

### Survival and prediction of outcome

During the follow-up period, 16 patients (24.6%) died at a mean age of 73.7 ± 0.7 years. The mean survival time after diagnosis was 3.6 ± 0.5 years. No significant baseline differences in ECG parameters between survivors and non-survivors were detected. However, non-survivors had: lower TDI-s (by −1.0 ± 0.4 m/s; *p* < 0.05), lower MDRD-estimated GFR (−19.2 ± 7.6; *p* < 0.05), higher hsTnT (difference 15.1 ± 2.5 pg/ml; *p* = 0.02). In univariate analyses, predictors of increased mortality included: higher heart rate (HR 1.041; *p* < 0.05), reduced renal function (MDRD) (HR 0.973; *p* < 0.05), elevated liver enzymes (HR 1.053; *p* < 0.05), longer QRS duration (as continuous variable) (HR 1.022; *p* < 0.05), increasing LV volumes (ESV, EDV), need for pacemaker implantation (HR 5.67; 95% CI 1.10–29.25; *p* < 0.05) (see Tables 5 and 6).

**Table 5:** Univariate survival analysis at baseline visit

Parameter	HR	95%-CI	*p*-value
**Demographics**			
Age Diabetes mellitus NYHA class BMI	1.0 8.8 2.6 1.1	O.9 - 1.1 1.5 - 49.8 0.9 - 7.9 0.9 - 1.2	n.s. *p* < 0.05 n.s. n.s.
***ECG***			
Heart rate QRS-duration	1.0 1.0	1.0 - 1.1 -	*p* < 0.05 n.s.
**Echocardiography**			
LV _mass_ Devereaux Stroke volume LV-EF MCF TAPSE Left atrium	1.0 1.0 1.0 0.0 0.4 1.0	- - - - -	n.s. n.s. n.s. n.s. n.s. n.s.
**Laboratory tests**			
hsTNT MDRD	1,0 1,0	- 0,9 - 1.0	n.s. *p* < 0,05

Hazard ratio (HR) and 95% Confidence intervals (CI) are depicted with their lower and upper limits of normal derived from the univariate analysis of the baseline visit. Cox-PH regression model. BMI: body mass index. LVmass Devereaux: left ventricular mass calculated according to the Devereaux formula indexed to the body surface area, LVEF: left ventricular ejection fraction, MCF: myocardial contraction fraction, TAPSE: tricuspid annular plane systolic excursion, hsTNT: high sensitivity troponin T, NT-proBNP: N-terminal pro brain natriuretic peptide, MDRD: modification of diet in renal disease, formula to calculate glomerular filtration rate

**Table 6:** Univariate survival analyses including baseline (BL) and follow-up (FU) visits

Parameter	HR	95%-CI	*p*-value
**Demographics**			
NYHA class BMI	0.4 1.0	0.1 - 1.2 1.0 - 1.0	n.s. n.s.
**ECG**			
QRS-duration	1.0	1.0 - 1.0	*p* < 0.05
**Echocardiography**			
IVS LV_mass_ Devereaux Stroke volume LVEF MCF TAPSE Left atrium	0.9 1.0 1.0 1.0 0.2 1.6 0.9	0.8 - 1.0 1.0 - 1.0 1.0 - 1.0 1.0 - 1.0 0.0 - 8.5 0.5 - 4.8 0.8 - 1.0	n.s. n.s. n.s. n.s. n.s. n.s. *p* < 0,05
**Laboratory test**			
MDRD	1.0	1.0 - 1.0	*n.s.*

Cox-PH regression models, Hazard ratio (HR), 95% confidence intervals (CI) with upper and lower limit of normal and p-values from the univariate analysis of differences between BL and one year FU visits are depicted. BMI: body mass index, IVS: intraventricular septum, LVmass Devereaux: left ventricular mass calculated from Devereaux-formula indexed to the body surface area. MCF: myocardial contraction fraction, TAPSE: tricuspid annular plane systolic excursion, MDRD: modification of diet in renal disease (formula for calculation of glomerular filtration rate)

Multivariate analysis confirmed renal function and liver enzymes as independent predictors of mortality. QRS duration showed prognostic association in univariate analysis but did not remain an independent predictor after adjustment.

Kaplan-Meier survival curve of QRS duration at baseline visits (BL) showed that patients presenting with low QRS duration ($\le$90 ms) presented a significantly better survival than patients with high QRS durations ( > 90 ms) (see Fig. 1D). At follow-up (FU) visits the effect was in trend also detectable (*p* = 0.14, see Fig. 1E). Here optimal cutoff for QRS duration was 154 ms. Interestingly, the Kaplan-Meier survival curve for patients presenting with a high delta QRS at FU visits compared to BL visits showed worse survival than patients without relevant QRS progression (see Fig. 2). Remarkably, patients who underwent a pacemaker implantation during the 12 month follow-up period presented in trend a worse survival than patients who did not need a pacemaker implantation (*p* = 0.062, see supplemental Fig. 1).

## Discussion

Cardiac ATTRwt amyloidosis is a cause of heart failure with increasing prevalence due to growing awareness and improvements in diagnostic tools, including bone scintigraphy, cardiac magnetic resonance imaging (MRI) and strain echocardiography. Recent consensus efforts, such as the French Delphi study on device therapy in cardiac amyloidosis, highlighted the clinical uncertainty surrounding electrophysiological aspects such as pacemaker or ICD implantation in these patients [18]. This reflects the current lack of evidence-based guidance for rhythm management in ATTRwt and underscores the need for reliable, non-invasive predictors of electrical disease progression and mortality.

Our longitudinal study of 65 treatment-naïve patients confirmed disease progression within 12 months, most prominently reflected in QRS duration and myocardial mass—both of which showed significant changes in the absence of causative therapy.

### Study cohort

Our analyses were conducted on a representative study cohort of ATTRwt amyloidosis patients from a German referral center. The comorbidities, sex distribution and prevalence of carpal tunnel syndrome in the medical history were suitable for the previously described study cohorts [4, 5, 7, 8, 19–24].

The mean survival time of patients with ATTRwt is three to four years [7, 8, 25]. This finding was consistent with the survival detected in this study cohort, which was 3.6 ± 0.5 years.

### Comparison of our data with established risk prediction parameters

#### Echocardiography

Thickening of the myocardial wall, especially the intraventricular septum (IVS) and posterior wall, is a well-known morphological sign of ATTRwt amyloidosis. This results in augmented myocardial mass [24, 26]. During our one-year follow-up period, the IVS diameter increased (1 mm ± 0.3), and the posterior myocardial wall diameter increased (0.7 mm ± 0.4). Therefore, detection of differences in echocardiography is near the detection threshold, hampered by the resolution of ultrasound images and intra- and interpersonal variances in image acquisition. Therefore, more sensitive parameters might be helpful for detecting disease progression within 12-month examination intervals.

#### Cardiac biomarkers

NTproBNP, the GFR and hsTNT are known to be sensitive and predictive risk biomarkers for myocardial amyloidosis. Recently, the predictive values of these parameters have been described in two broad independent study cohorts in the U.S. and the U.K. [6, 7]. These studies identified NTproBNP, hsTNT and the GFR as suitable risk prediction models for ATTR amyloidosis. Furthermore, elevated NTproBNP and hsTNT levels indicate increased four-year mortality [7].

A common weakness of these serological parameters is that they vary depending on renal function as well as de- and recompensation status. Therefore, these parameters tend to differ intrapersonally, rendering decisions about stable or progressive disease difficult during two examinations, especially at shorter examination intervals. However, as specific disease-modifying therapies for cardiac ATTR cardiomyopathy are cost intensive, early decisions about effectiveness and the need for a switch in drug class are important to achieve the most appropriate cost benefit efficiency.

In our study, a correlation between survival and renal function was confirmed, and non-survivors presented a significantly lower MDRD than survivors did. Furthermore, patients who did not survive the one-year follow-up period presented more severe cardiac involvement than survivors did. In contrast to previous studies with longer follow-up, which reported a steady increase in cardiac biomarkers alongside disease progression [7], we did not find significantly differences in levels of hsTNT and NTproBNP at the 12-month follow-up compared to baseline in this study. This finding underlines the need for additional markers to identify patients with progressive disease and elevated mortality risk.

Therefore, evaluation of the QRS duration might add an interesting objective parameter, with reduced intrapersonal variances. In addition it offers the possibility of automated detection by broadly available ECG devices. Therefore, we analyzed disease progression with a focus on detailed analyses of ECG findings.

### Additive value of QRS complex progression in ATTRwt patients

The need for pacemaker implantation is associated with a higher incidence of mortality [8]. In addition, in this study, the progression of the QRS duration and reduction of EF were indicators of the progression of heart failure and worse survival. During follow-up, abnormal ECGs, including bundle branch block and prolonged QRS duration, which are indicators of progressive disease, were more prevalent.

ECG findings in ATTR cardiomyopathy have been described by several groups. In particular, low-voltage patterns, bundle branch blocks and pseudoinfarction patterns have been described in ATTRv and ATTRwt [8, 24, 27]. However, to our knowledge, no information about the prognostic impact of QRS duration on ATTRwt exists.

Interestingly, in our study cohort, the QRS complex duration was significantly longer at follow-up examinations than at baseline. Furthermore, a positive correlation between QRS- and QTC-prolongation in ECG and myocardial mass, a negative correlation between TAPSE and QRS duration and a positive correlation between hsTNT levels, QRS complex duration and myocardial mass were reported. At follow-up, the QRS duration was correlated with intraventricular septum thickness, stroke volume and hsTNT levels.

Taken together, these findings indicate a correlation between QRS duration and LV function and support our suspicion that QRS duration could be a very important parameter for identifying progressive cardiac ATTRwt amyloidosis.

In summary, a correlation between QRS duration and the severity of cardiac amyloidosis was shown. Hereby, the severity of cardiac amyloidosis is depicted by morphological and functional echocardiographic as well as laboratory parameters.

### Confounding factors affecting QRS duration

For this study, the ECGs were conducted by a dedicated and standardized ECG department using consistent lead placement protocols. This ensures standardized recordings and reproducible results. Nonetheless, clinical confounders must be acknowledged. Studies on reproducibility of ECGs have confirmed that QRS duration is a largely stable value and changes in electrode positions results in only minimal measurement inaccuracies. Namely, QRS duration varies between 0.05–1.15 ms in case of minor ECG electrode position changes of less than 2 cm [28]. These values are thus far below the difference detected in this study. Thus, methodological inconsistencies are unlikely to explain the documented QRS progression.

Furthermore, medications such as diuretics, beta-blockers and antiarrhythmics can affect QRS morphology and duration by influencing conduction velocity and heart rate.

After the baseline visit, patients received recommendations for adjusting their heart failure therapy according to the ESC recommendations for amyloidosis [17]. Accordingly, diuretics are regularly adjusted under laboratory controls according to need and kidney function by the general practitioner. Beta blockers are recommended to reduce in cases of advanced amyloidosis, since patients with this condition primarily increase their cardiac output via heart rate. Digitalis is not recommended for patients with amyloidosis; however, amiodarone is often used to control atrial fibrillation frequency.

Amiodarone primarily affects repolarization and atrioventricular (AV) conduction time. At normal heart rates, it does not significantly affect QRS time [29]. Since this study cohort had normal heart rates and no significant changes were detected during follow-up examinations, any effect on QRS duration is negligible. At baseline visits heart rate was 73.5 bpm ±1.7 and 2.1 ± 1.8 bpm higher an follow-up visit.

Loop diuretics may potentially indirectly prolong QRS via electrolyte shifts, but only under severe hypo- or hyperkaliemia which was not the case during the study visits [30].

## Conclusions and limitations

In conclusion, this study revealed that worse survival is associated with a decrease in cardiac function and that worsening renal function and disease progression are associated with the need for pacemaker implantation and increased QRS duration. Surprisingly, NTproBNP and hsTNT could not identify patients with worse survival in this study cohort during the short-term follow-up period of 12 months, which might be due to the short follow-up periods and its dependency on renal function.

The established risk prediction models use laboratory markers, mainly NTproBNP, hsTNT and the GFR, to predict outcomes in ATTRwt patients [6, 7], which are dependent on volume status, renal function and intraindividual variation from visit to visit. Furthermore, the calculation of myocardial thickness is not very sensitive, as small variances in image acquisition may result in varying measurements of wall thickness. In contrast, QRS duration is calculated automatically via broadly available ECG devices, which could be valuable for detecting subtle changes in QRS duration. This could facilitate the early identification of progression in patients with ATTRwt amyloidosis, which could inform decisions regarding the prescription of expensive, specific medications. However, broader studies are needed to confirm its sensitivity and predictive value for outcomes in ATTRwt patients.

## Electronic supplementary material

Below is the link to the electronic supplementary material.

Supplementary Material 1

## Data Availability

All data generated or analyzed during this study are included in this published article.
