# Supplementary material for: Progression of QRS duration – a potential surrogate marker of survival in ATTRwt amyloidosis patients
Source: Orphanet J Rare Dis. 2025 Oct 20;20:523. doi: 10.1186/s13023-025-04078-4 (PMC12539068; doi:10.1186/s13023-025-04078-4)
Supplement: Supplementary file 1 — Supplementary Material 1 [file 13023_2025_4078_MOESM1_ESM.pptx]

## Slide 1
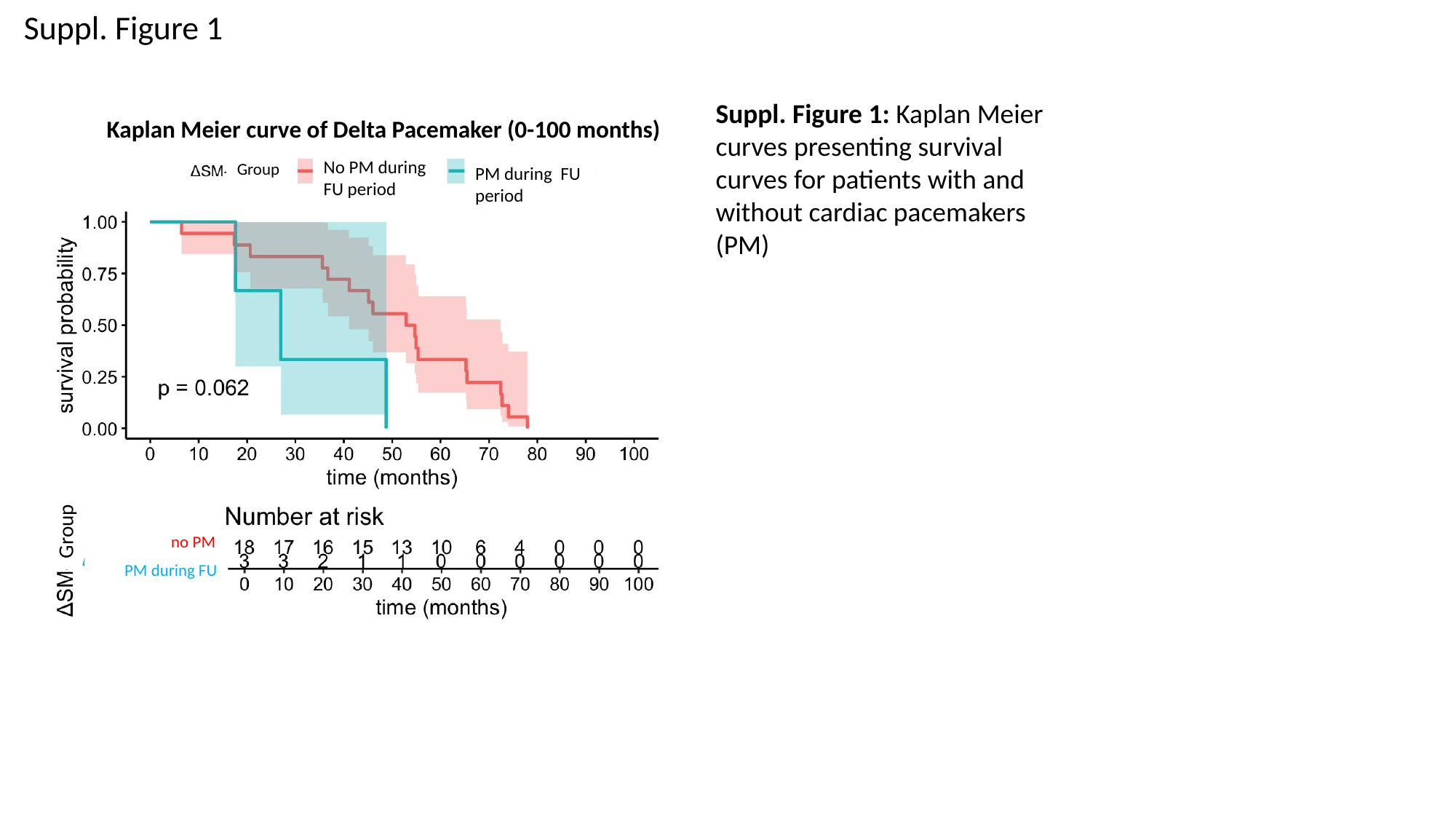

Suppl. Figure 1
Suppl. Figure 1: Kaplan Meier curves presenting survival curves for patients with and without cardiac pacemakers (PM)
Kaplan Meier curve of Delta Pacemaker (0-100 months)
No PM during
FU period
Group
PM during FU period
Group
no PM
 PM during FU
